# Percutaneous laser ablation for benign thyroid nodules: a meta-analysis

**DOI:** 10.18632/oncotarget.17928

**Published:** 2017-05-17

**Authors:** Wei Fan Sui, Jian Yun Li, Jian Hua Fu

**Affiliations:** ^1^ Department of Interventional Radiology, The Affiliated Renmin Hospital of Jiangsu University, Zhenjiang, 212002, China

**Keywords:** percutaneous laser ablation, benign thyroid nodules, meta-analysis

## Abstract

**Purpose:**

To evaluate percutaneous laser ablation in treating benign thyroid nodules, we conducted a meta-analysis based on summarizing existing researches.

**Materials and Methods:**

A literature search for clinical trial was performed in PubMed, Cochrane Library and Excerpt Medica Database. The qualities of included studies were evaluated. We calculated the indexes with mean difference. Heterogeneity and publication bias were tested and explored. We performed subgroup analyses and sensitivity analysis further.

**Results:**

A total of 19 researches and 2137 patients were included in this meta-analysis. The pooled estimates of nodule volume were statistically significant after percutaneous laser ablation for 1 month, 3 month, 6month, 12month, 24month and 36month(*P* < 0.05). The pooled estimate of thyroid-stimulating hormone was statistically significant after percutaneous laser ablation for 1 and 12 month (*P* = 0.008 and *P* = 0.03). The pooled estimate of free triiodothyronine was no statistically significant after percutaneous laser ablation for all follow-up intervals. The pooled estimate of free tetraiodothyronin was statistically significant after percutaneous laser ablation1 month (*P* = 0.004). The pooled estimate of thyroglobulin was statistically significant after percutaneous laser ablation 24 month (*P* = 0.04). The heterogeneity was found and the source of heterogeneity was explored in nodule volume for 6 and 12 month. No publication bias was found.

**Conclusions:**

This meta-analysis demonstrated that percutaneous laser ablation was safe and useful in shrinking benign thyroid nodules volume, improving thyroid function, relieving symptoms of pressure and esthetic, especial for hyper-vascular benign thyroid nodules. Larger number of high-quality prospective studies still needs to be performed.

## INTRODUCTION

Thyroid nodules (TNs) are frequently seen in adults with a prevalence of approximately 20% to 76% in the general population [1–2]. Although TNs are mostly benign, there exists a possibility for malignant transformation [3]. Benign TNs may be treated due to secondary issues such as dyspnea and hoarseness associated with the location of the nodules, or cosmetic concerns of patients [4]. Surgery is the standard therapy for treatment of TNs, but it requires general anesthesia and may lead to scar formation and may not be an option for all patients, depending on other health conditions [5]. Alternative non-invasive treatments such as levothyroxine therapy and radioiodine therapy have been proposed for older individuals or those with high surgical risk; however, these treatments can also cause iatrogenic hypothyroidism or hyperthyroidism [6, 7]. Therefore, invasive image-guided ablation therapies including ethanol ablation, radiofrequency ablation (RFA) and laser ablation (LA) have been used and proven clinically effective [8–10].

Ethanol ablation is used to shrink the volume of cystic TNs; however, the complications of ethanol seepage and subsequent injury to the adjacent tissue limit its clinical utility [11, 12]. RFA is a thermal ablation technique that has been investigated in patients with benign TNs. Previous meta-analyses of RFA and several other studies have demonstrated that RFA can improve outcomes and prognosis of benign TNs [7, 12–14]. Moreover, recently published studies have suggested that RFA can be recommended as a first-line therapy [15]. However, the potential adverse effects of RFA cannot be ignored and include pain, hematoma formation and fever. Guidelines suggest that RFA is not currently recommended as a first-line therapy for benign TNs [2]. The clinical utility of RFA for the treatment of benign TNs remains controversial. Interstitial laser photocoagulation (LP) or percutaneous laser ablation (PLA) are other thermal ablation techniques that are alternative treatments for individuals with benign TNs and high surgical risk [16, 17]. However, the existing studies report varied results. Guidelines suggest that only experienced doctors should conduct LP [2].

Systematic review and meta-analysis are widely accepted for summarizing current evidence-based research to update treatment guidelines. To our knowledge, no one has conducted a meta-analysis regarding the use of LP to treat benign TNs. We performed a systematic review and meta-analysis to assess the clinical value of LP or PLA in the treatment of benign TNs, recording information regarding observed indices including the volume of nodules and levels of thyroid-stimulating hormone (TSH), free triiodothyronine (fT3), free tetraiodothyronine (T4) and thyroglobulin (Tg).

## RESULTS

### Literature search

**Figure 1:**
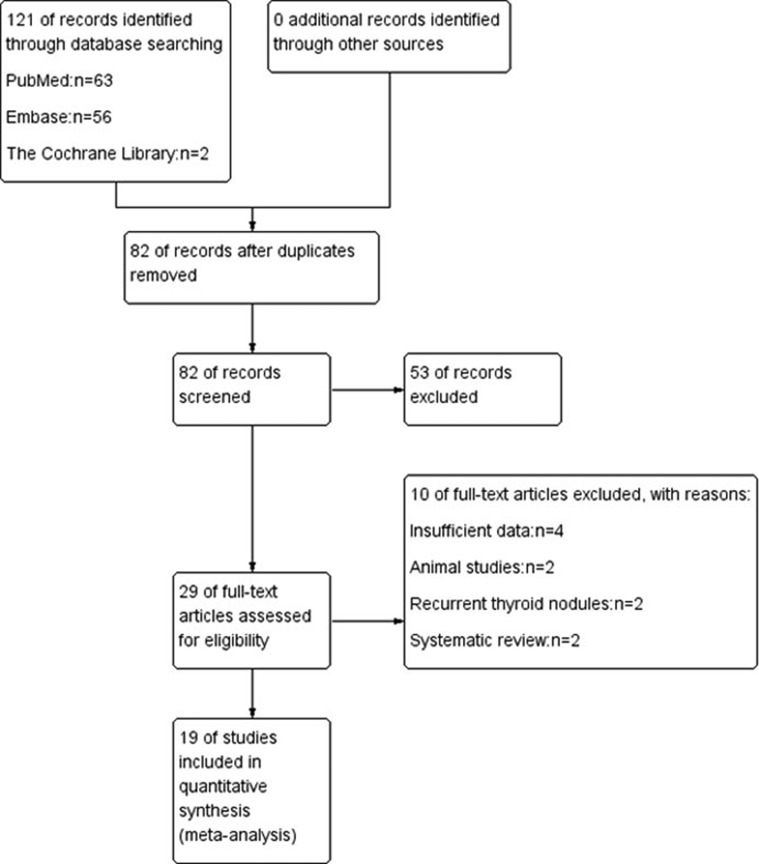
Flowchart shows flow of information through the different phases of systematic review toward a meta-analysis

Originally, a total of 121 records were found (63 in PubMed, 56 in Embase and 2 in The Cochrane Library). Then, we narrowed the scope of the records after browsing the titles. After perusing the abstract and full text of the remaining records, we excluded 2 animal studies, 2 of studies recurrent thyroid nodules, 2 systematic reviews and meta-analyses, and 4 studies with insufficient data (Figure 1). We ultimately selected 19 studies for this meta-analysis [25–43].

### Data extraction and quality assessment

The extracted data contained information including the author, publication year, country, study design, number of patients, patients’ age, TN characteristics, laser energy and times, observed indices, and follow-up interval (Supplementary Table 1). Many of the included studies were performed in Denmark or Italy, followed by Turkey and Egypt. Four prospective studies, six retrospective studies and nine NG studies were included. Almost all TNs in the included studies were benign thyroid nodules. The observed indices were nodule volume, TSH, T3, T4 and Tg, as well as TPOAb, TgAb, anti-TPOAb and anti-TPO. The follow-up intervals were 1 to 48 months.

**Figure 2:**
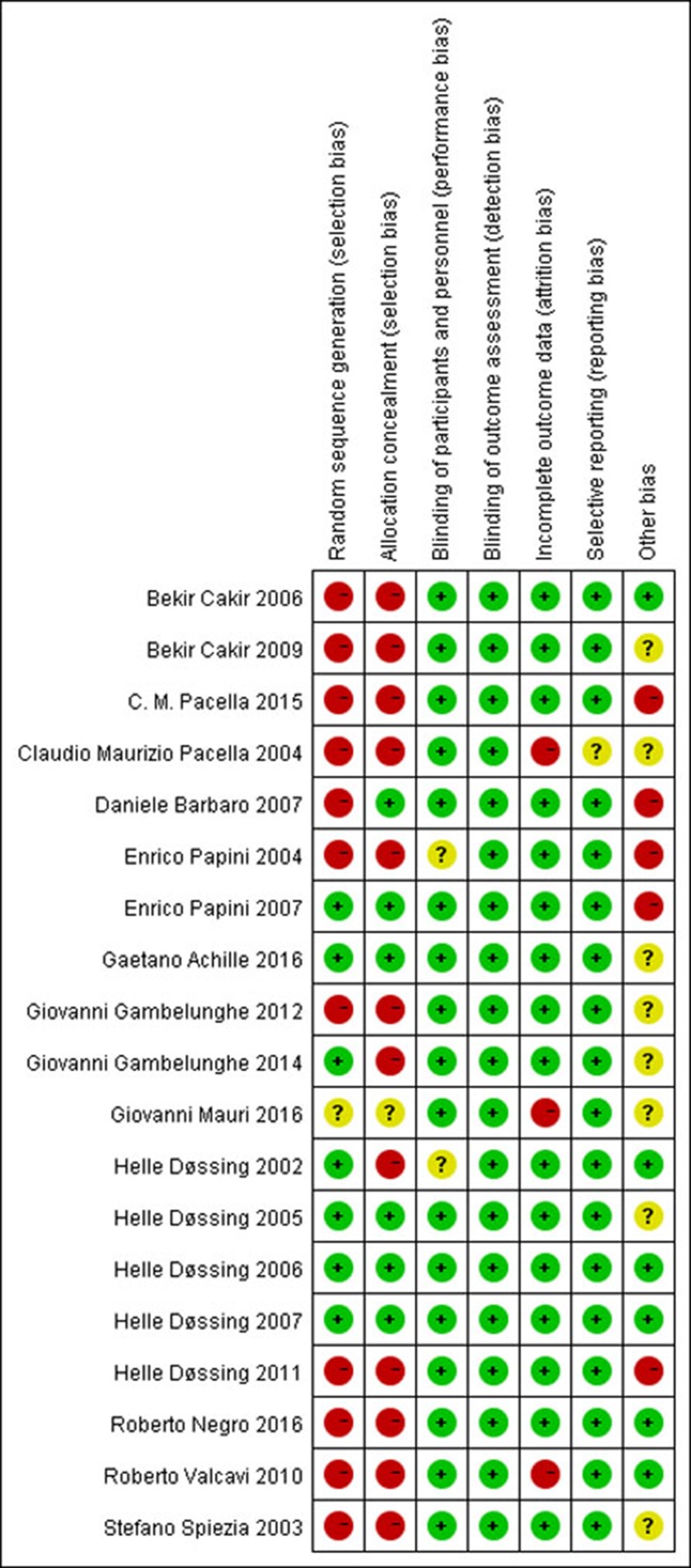
Risk of bias summary: review authors’ judgements about each risk of bias item for each included study − high risk, +: low risk,?: unclear risk.

**Figure 3:**
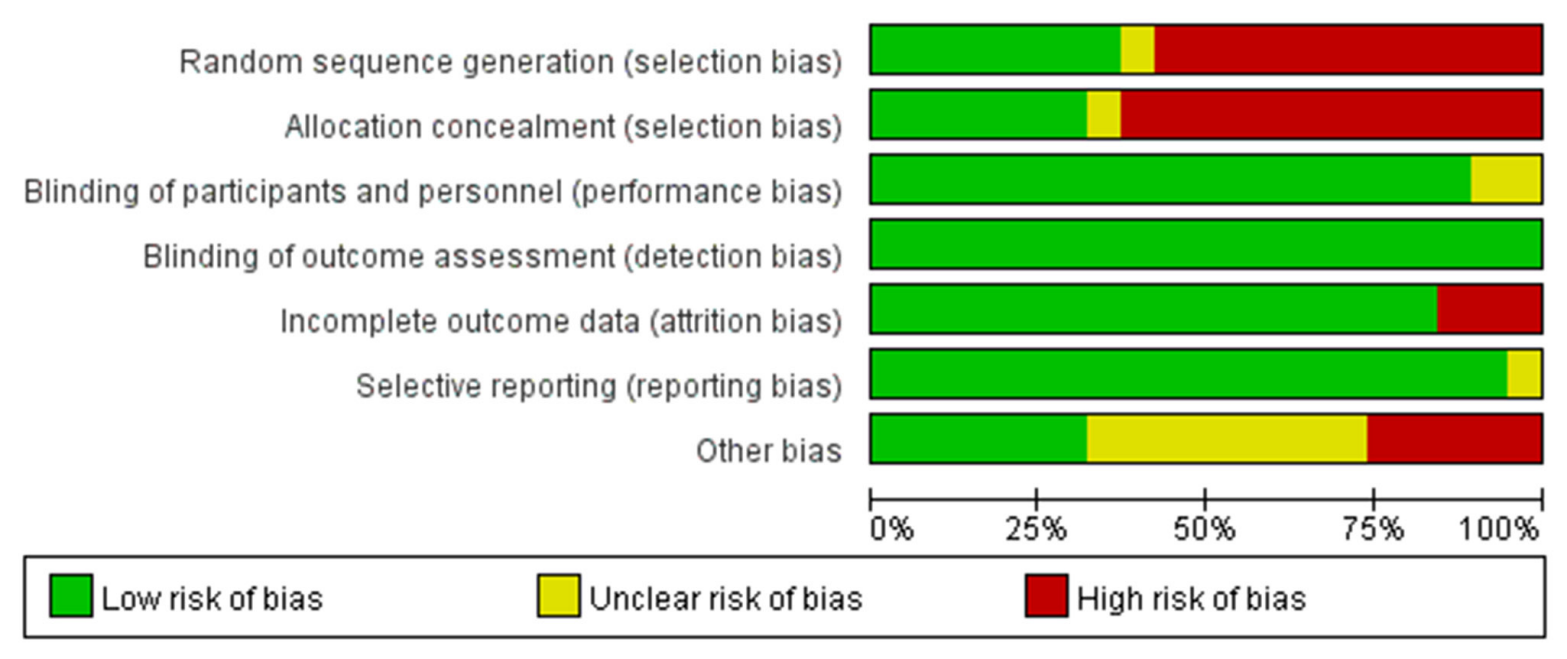
Risk of bias graph: review authors' judgements about each risk of bias item presented as percentages across all included studies

The risk of bias using the Cochrane Collaboration’s tool was performed to assess the quality of the included studies. It contains seven bias metrics including random sequence generation (selection bias), allocation concealment (selection bias), blinding of participants and personnel (performance bias), blinding of outcome assessment (detection bias), incomplete outcome data (attrition bias), selective reporting (reporting bias), and other bias. The investigators as low risk, unclear risk or high risk, represented as green, yellow and red colors, respectively, evaluated bias. The summary and graphs of the risk of bias were constructed based on the investigators’ judgments about each risk of bias item for each included study and represented as percentages across all the included studies (Figures 2, 3).

### Data analysis

#### Observed index and heterogeneity test

**Figure 4:**
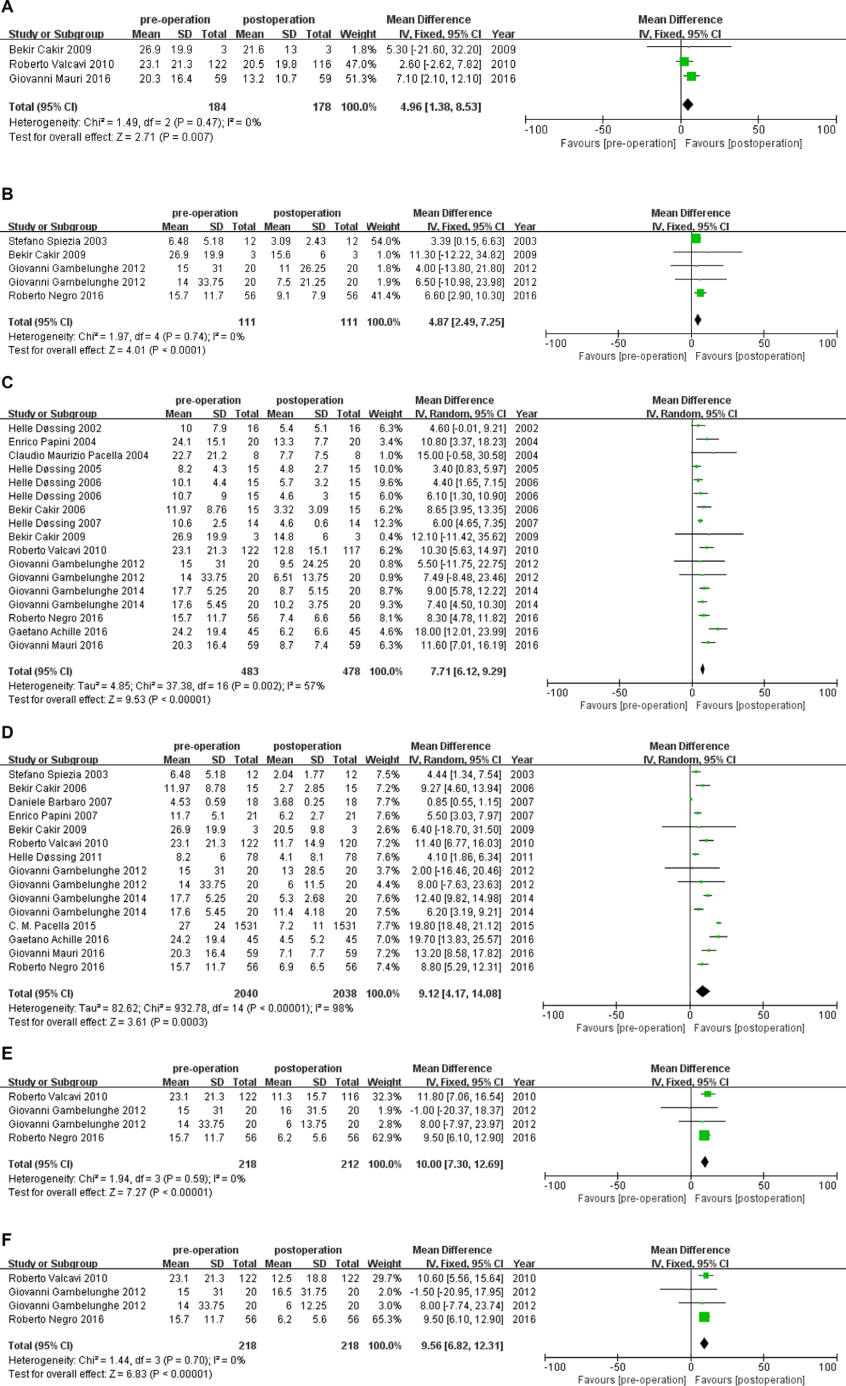
(**A**–**F**) Volume changes of benign TNs before, 1,3 ,6, 12, 24 and 36month after PLA treatment, respectively. PLA= Percutaneous Laser Ablation, CI = confidence interval, TN = thyroid nodule.

The included studies compared the changes in nodule volume in 1, 3, 6, 12, 24 and 36 months after PLA treatment compared to baseline (Figure 4A–4F). The changes in nodule volume after PLA treatment were statistically significant for all intervals (1 month, MD 95% CI : 4.96 [1.38,8.53], *P =* 0.007; 3 month, MD 95% CI : 4.87 [2.49,7.25], *P <* 0.007; 6 months, MD 95% CI : 7.71 [6.12,9.26], *P <* 0.00001; 12 months, MD 95% CI : 9.12 [4.17,14.08], *P =* 0.0003; 24 months, MD 95% CI : 10 [7.30,12.69], *P <* 0.00001; 36 months, MD 95% CI : 9.56 [6.82,12.31], *P <* 0.00001). This indicates that PLA reduced the size of benign TNs effectively. For heterogeneity assessment, the I^2^ value for nodule volume for 1, 3, 6, 12, 24 and 36 months was 0%, 56%, 57%, 98%, 0% and 0%, respectively. This indicates that no heterogeneity existed in nodule volume change at 1, 24 and 36 months, moderate heterogeneity existed in nodule volume change at 3 and 6 months, and significant heterogeneity existed in nodule volume change at 12 months. The source of heterogeneity at 6 and 12 months post-PLA treatment was further explored using subgroup analysis.

**Figure 5:**
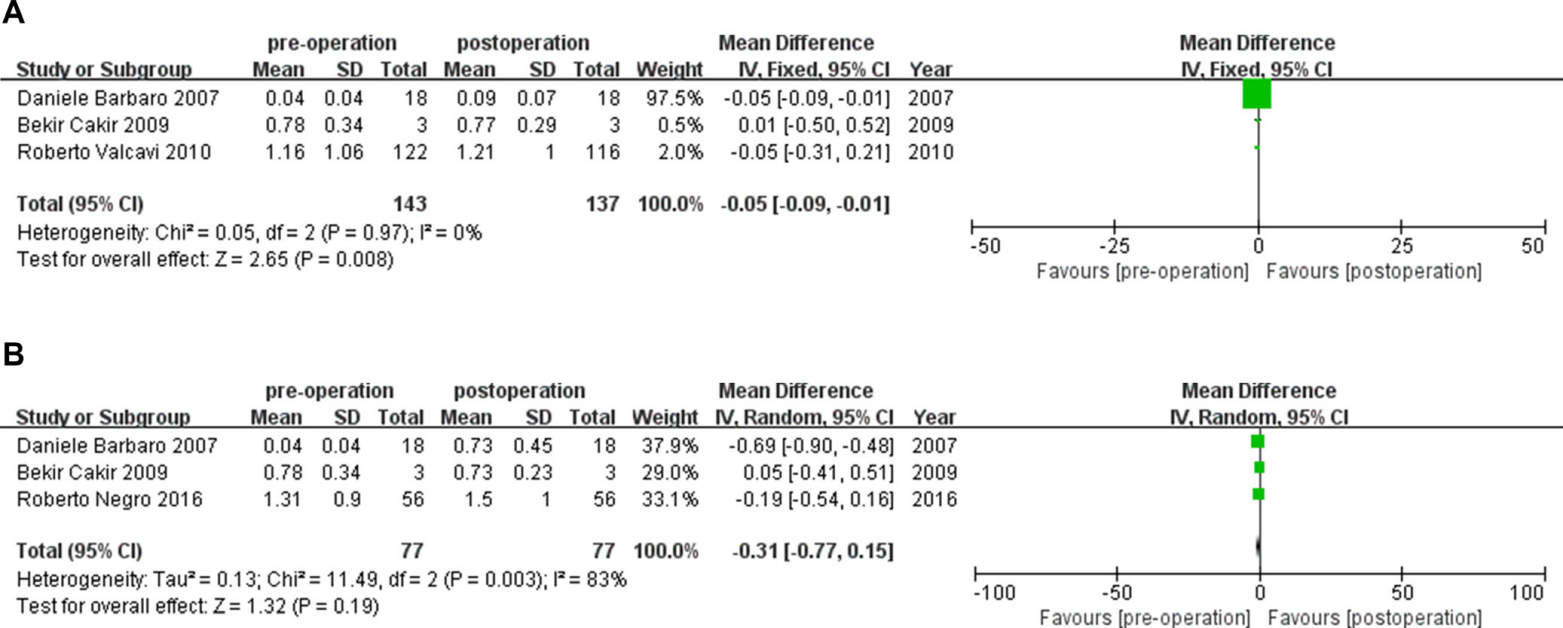
(**A**–**B**) TSH changes of benign TNS before, 1, 3month after PLA treatment, respectively. PLA= Percutaneous Laser Ablation, CI = confidence interval, TN = thyroid nodule.

We compared the changes in TSH levels at 1, 3, 6, 12, 24 and 36 months after PLA treatment with baseline values. The TSH level after PLA was statistically significant for 1 and 12 months (Figure 5A, 5B) (1 month, MD 95% CI: -0.05 [−0.09, −0.01], *P =* 0.008; 12 months, MD 95% CI: −0.25 [−0.47, −0.03], *P =* 0.03) and was not statistically significant for 3, 6, 24, 36 months (3 months, MD 95% CI: −0.31 [−0.77,0.15], *P =* 0.19; 6 months, MD 95% CI: 0.29 [−0.18,0.75], *P =* 0.22; 24 months, MD 95% CI: 0.01 [−0.16,0.17], *P =* 0.94; 36 months, MD 95% CI : 0.02 [−0.14,0.19], *P =* 0.78). This indicates that increased serum TSH levels were present in patients with benign TNs after treatment with PLA at 1 and 12 months. For heterogeneity assessment, the I^2^ value for TSH level at 1 and 12 months post−PLA treatment was 0% and 71%, respectively. This indicates that no heterogeneity existed in TSH levels at 1 month and moderate heterogeneity existed in TSH levels at 12 months. However, the number of included studies that reported TSH levels at 12 months was less than 10 studies [19, 20]; therefore, the source of heterogeneity for this value was not explored further.

Compared with baseline levels, the change in T3 levels after treatment with PLA was not statistically significant (1 month, MD 95% CI: 0.19 [−0.24,0.63], *P =* 0.38; 3 months, MD 95% CI: 0.34 [−0.25,0.92], *P =* 0.26; 6 months, MD 95% CI: −0.27 [−0.61,0.08], *P =* 0.14; 12 months, MD 95% CI: 0.25 [−0.08,0.59], *P =* 0.13; 24 months, MD 95% CI: 0.04 [−0.22,0.30], *P =* 0.77; 36 months, MD 95% CI: 0.06 [−0.04,0.16], *P =* 0.21). This indicates that PLA had no effect on the serum T3 levels in patients with benign TNs. Heterogeneity assessment was not performed as there was no statistical significance in T3 levels post-PLA treatment.

**Figure 6:**
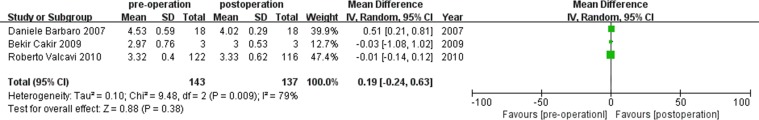
T4 changes of benign TNS before and 1 month after PLA treatment PLA= Percutaneous Laser Ablation, CI = confidence interval, TN = thyroid nodule.

The change in T4 levels after PLA treatment was statistically significant at 1 month (MD 95% CI: −0.21 [−0.03, −0.09], *P =* 0.004) when compared with baseline (Figure 6) and not statistically significant for the remaining intervals (3 months, MD 95% CI: 0.06 [−0.30,0.4], *P =* 0.75; 6 months, MD 95% CI : −0.27 [−0.61,0.08], *P =* 0.14; 12 months, MD 95% CI : 0.34 [−0.18,0.87], *P =* 0.20; 24 months, MD 95% CI : 0.29 [−0.38,0.96], *P =* 0.40; 36 months, MD 95% CI : 0.38 [−0.36,1.12], *P =* 0.31). This indicates that PLA was able to reduce serum T4 levels in patients with benign TNs at 1 month post-PLA treatment. For heterogeneity assessment, the I^2^ value for serum T4 levels at 1 month was 44%. This indicates that moderate heterogeneity exists in serum T4 levels at 1 month post-PLA treatment. The source of heterogeneity in serum T4 levels at 1 month was not explored further as the number of included studies that reported this observed index was less than 10 studies [20, 21].

**Figure 7:**
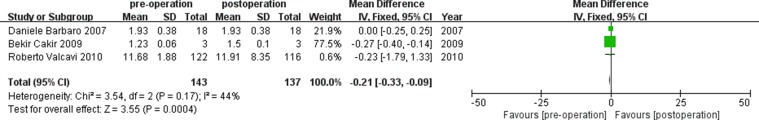
Tg changes of benign TNS before and 1 month after PLA treatment PLA = Percutaneous Laser Ablation, CI = confidence interval, TN = thyroid nodule.

The change in Tg levels between baseline and post-PLA treatment was also assessed. The change in Tg after PLA treatment was statistically significant at 24 months (Figure 7) (MD 95% CI : 12.30 [0.56,24.03], *P =* 0.04) and not statistically significant for the remaining intervals (1 month, MD 95% CI : −34.05[−87.37,19.28], *P =* 0.21; 3 months, MD 95% CI : −8.9 [−58.36,40.56], *P =* 0.72; 6 months, MD 95% CI : 0.06 [−10.45,10.58], *P =* 0.99; 12 months, MD 95% CI: −14.03 [−30.34,2.38], *P =* 0.09; 36 months, MD 95% CI : 1.72 [−39.98,43.41], *P =* 0.94). For heterogeneity assessment, the I^2^ value for Tg at 24 months was 0%, and therefore, no source of heterogeneity was explored.

### Meta-regression analysis and subgroup analysis

Subgroup analysis was performed to explore the source of heterogeneity rather than meta-regression analysis due to the use of RevManManger 5.3, which cannot perform such analysis. Based on the heterogeneity test previously described, the source of heterogeneity for nodule volume after PLA treatment at 6 and 12 months was conducted (Table 1).

**Table 1:** Subgroup analyses percutaneous laser ablation in treating benign thyroid nodules in 6 and 12 month

Subgroup	Studies	MD (95% CI)	Tau^2^	Chi^2^	I^2^	*P*
**Nation**						
**6 month**						
Asian-Africa	3	8.48 [5.68, 11.27]	0	0.11	0	0.95
European	11	6.43 [5.56, 7.31]	2.69	22.42	46%	0.03
**12 month**						
Asian-Africa	3	8.94 [6.15, 11.72]	0	0.06	0	0.97
European	11	2.23 [1.95, 2.50]	76.4	930.28	99%	< 0.0001
**Design**						
**6 month**						
prospective	4	5.28 [4.24, 6.32]	0	3.74	0	0.44
retrospective	6	9.40 [7.86, 10.93]	3.4	11.46	39%	0.12
**Nodule type**						
**6 month**						
functional	3	6.22 [4.90, 7.54]	4.73	2.78	28%	0.25
Non-functional	11	7.07 [5.99, 8.16]	7.17	33.63	61%	0.001
**Energy**						
**6 month**						
Less 1000J	3	7.46 [5.53, 9.39]	0	2.41	0	0.66
between1000J and 2000J	2	15.16 [10.50, 19.83]	14.7	2.19	54%	0.14
between 2000J and 3000J	5	4.74 [3.11, 6.38]	0.45	4.45	10%	0.35
more than 3000J	4	9.85 [7.48, 12.22]	0	1.76	0	0.62
**12 month**						
less than 1000J	2	9.66 [7.72, 11.59]	13.44	10.10	70%	0.02
between 1000J and 2000J	4	15.25 [14.18, 16.33]	87.86	154.33	98%	< 0.0001
between 2000J and 3000J	3	5.19 [3.67, 6.72]	4	3.31	39%	0.19
more than3000J	3	10.67 [8.28, 13.06]	0.79	2.34	15%	0.31

In the subgroup analysis for nodule volume after PLA treatment at 6 months, the I^2^ values for Asia/Africa, Europe and the total for both continents were 0%, 46% and 39%, respectively, indicating that continent was a source of heterogeneity. The I^2^ values for prospective studies, retrospective studies and the total for both types of study were 0%, 39% and 65%, respectively, indicating that design was a source of heterogeneity. The I^2^ values for functional TNs, non-functional TNs and the total for both types of TNs were 28%, 67% and 51%, respectively, indicating that nodule type was a source of heterogeneity. The I^2^ values for energy levels less than 1000J, between 1000J and 2000J, between 2000J and 3000J, more than 3000J and the total for all energy levels were 0%, 54%, 10%, 0% and 58%, respectively, indicating that energy level was a source of heterogeneity.

Subgroup analysis for nodule volume after PLA treatment at 12 months was performed only for continent and energy level due to a lack of prospective studies and only a single functional TN reported. The I^2^ values of Asia/Africa, Europe and the total for both continents were 0%, 99% and 98%, respectively, indicating that continent was a source of heterogeneity. The I^2^ values of energy levels less than 1000J, between 1000J and 2000J, between 2000J and 3000J, more than 3000J and the total for all energy levels were 70%, 98%, 39%, 15% and 95%, respectively, indicating that energy level was a source of heterogeneity.

### Sensitivity analysis and publication bias

Sensitivity analysis was performed to consider the stability of the results. We found no significant changes with removing any one study. To assess for publication bias, funnel plots and Egger’s linear regression were conducted and no publication bias was found.

## DISCUSSION

This meta-analysis demonstrates that PLA has significant clinical value in the treatment of benign TNs for reducing nodule volume after 1, 3, 6, 12, 24 and 36 months. PLA is an effective technique for improving thyroid function, including increasing serum TSH levels and reducing serum T4 and Tg levels at 1 and 12 months post-treatment. Subgroup analyses revealed that continent, study design, types of TNs and energy levels were significant factors for research assessing the usefulness of PLA in the treatment of TNs. Type of TNs and energy levels were the most significant factors in assessing the effectiveness of PLA in treating TNs.

In principle, PLA can lead to irreversible cell damage and the formation of thermal coagulation necrosis with high temperatures through directly transmitting laser light into the target tissue via flexible fibers [44, 45]. The clinical effectiveness of PLA in reducing the TN volume was evaluated by performing *ex vivo* and *in vivo* studies in animals [46, 47] as well as numerous clinical studies [25–43]; however, previous research had the drawback of uniform follow-up intervals. In this meta-analysis, we summarize previous outcomes and demonstrate short and long term efficacy in decreasing the size of TNs with PLA treatment at 1, 3, 6, 12, 24 and 36 months post-treatment reported in terms of statistics (*P <* 0.05). Based on the summarized outcomes at 6 and 12 months, we found heterogeneity among the results. Therefore, subgroup analyses were conducted to explore the source of heterogeneity and try to explain it. Continent, study design, nodule type and energy level were identified as the sources of heterogeneity at 6 months post-PLA treatment. Continent and energy level were identified as the sources of heterogeneity at 12 months post-PLA treatment.

Further epidemiological studies are indicated to explore the significance of continent on the treatment of TNs, including nodule reduction and functional thyroid changes after PLA. Energy level was another source of heterogeneity. Gambelunghe G [38] and Ritz J [44] demonstrated that high energy levels (> 500J) had a positive correlation with nodule volume reduction in short and long follow-up intervals after PLA. However, they also found that high-energy levels were associated with an increased complication rate after PLA. Our meta-analysis indicates that low and high energy levels all had a positive effect on reducing nodule volume. Moreover, several studies [27, 36, 42, 49] with ultra-high energy levels (> 3000J) achieved a reduction in benign TN size of approximately 50% with few side effects or failures. Laser fiber delivers energy to the target more accurately and limits energy in the target more than RF electrodes. Through high- frequency alternating current, RF electrodes generate heat and create a close-loop circuit resulting in energy deposition into the patient’s body. In terms of energy availability, PLA is more efficacious than RFA [42] and previous research has demonstrated the clinical significance of PLA in the treatment of metastatic lymph nodes in the neck secondary to thyroid carcinoma [50]. Further research on high-energy options for managing benign TNs with varied nodule types as well as adverse events associated with these options is indicated. Nodule type was also regarded as a source of heterogeneity in our meta-analysis. Previous research has reported that the vascularity of TNs may be linked with nodule reduction and even thyroid function and found that PLA and RFA had better effects in treating hyper-vascular TNs [6, 32, 36]. Consequently, vascularity of TNs may explain why nodule type was regarded as a source of heterogeneity and hyper-vascular TNs may be treated more successfully than other types of TNs. In regard to nodule types, the serum levels of TSH, T3, T4 and Tg cannot be ignored. We found increased TSH levels, no change in T3 levels, and reductions in T4 and Tg levels. The destruction of TNs by PLA leads to decrease in hormones such as T3 and/or T4, which in turn accelerate secretion of TSH by the pituitary. However, the reduced serum T3 level was inconsistent with unchanged serum T4 level in this meta-analysis. Tg level reflects thyroid mass, injury and TSH receptors [48]. The decreased nodule volume may be connected to the decreased serum Tg levels after PLA, which has also been shown in research on RFA [49]. The serum level may not be the indicator after PLA and RFA expect hyper-vascular TNs.

There were no major complications found, and only a few patients had minor complications according to the definition provided by the Society of Interventional Radiology [51, 52] of PLA in previous research [25–43]. Due to a lack of sufficient data, we were unable to perform further analysis to elucidate the relationship between complications and PLA treatment. However, previous research has not found any significant relationship between complications and PLA or RFA in treating TNs [4, 6, 7, 15]. Off-target effects have, however, been reported in previous research on the treatment of liver disease with RFA [53, 54]. Ontogenesis is a possible off-target effect attributed to c-met/hepatocyte growth factor axis alterations leading to a reversal in liver regeneration after RFA [53]. Fortunately, such off-target effects have not been reported in the treatment of TNs by PLA or RFA. We still are unable to affirm, however, that PLA is safer than RFA as a treatment for TNs. Further study regarding off-target effects and similar complications related to PLA in the treatment of TNs is necessary. Several included studies unsurprisingly demonstrated [25, 29, 37] that PLA could reduce the volume of TNs and relieve symptoms of pressure and improve aesthetics in both the short and long term. Døssing H also purported [31] that a series of three PLA treatments yielded better relief of pressure and aesthetic symptoms than one treatment alone. Therefore, PLA was determined to be both safe and useful in relieving symptoms of pressure and improving aesthetics.

There were some limitations in our meta-analysis. 1) There were not enough RCTs included in the selected studies and data from some excluded studies could not be extracted, which may induce bias and affect our assessment of management of TNs after PLA. 2) There was a lack of sufficient statistical data to demonstrate whether more than one treatment of PLA was better than just one treatment PLA in treating TNs and to demonstrate the relationship between nodule volume and optimal energy selection. 3) Meta-regression analysis to explore the source of heterogeneity could not be performed due to the use of RevManManger 5.3.

In conclusion, PLA is a promising technique to treat benign TNs, especially hyper-vascular TNs. This technique has been shown to reduce nodule volume, improve thyroid function, relieve symptoms of pressure, improve aesthetics and is safer with fewer complications and expenses than RFA. Further high-quality prospective studies regarding the use of PLA in managing TNs still need to be performed.

## MATERIALS AND METHODS

This research was published according to PRISMA guidelines [18], and neither outside ethical approval nor patient written informed consent was necessary due to the characteristics of systematic review and meta-analysis. Two investigators conducted all the intermediate steps individually, and all three investigators performed a final review.

### Search strategy

To find all relevant published studies, we identified the following research question: what is the clinical significance of PLA in the treatment of benign thyroid nodules? The scope of the search was then conducted with respect to this question.

We performed a comprehensive literature search using PubMed, Cochrane Library and Excerpt Medica Database up until November 30, 2016. We used the following search terms in the field for Title/Abstract and/or keywords: “interstitial laser photocoagulation”, “percutaneous laser ablation”, “benign”, and “thyroid nodules”. All the data were available from published papers without restriction.

### Study selection

The studies selected were required to meet the following inclusion criteria: a) The original research. b) The study participants were human. c) The study compared clinical results such as TN volume, TSH, T3, T4 and Tg both pre-operatively and post-operatively, as well as compared these values among patients who were treated with LA to those treated with other therapies. 4) The study demonstrated the clinical value of percutaneous laser ablation for benign thyroid nodules.

### Data extraction and data quality assessment

Two investigators extracted information regarding the author, nation, published year, study design, and population characteristics, including the number of patients, patients’ ages and characteristics of patients’ thyroid nodules, complications, instrumental parameters, observed indices including nodule volume, TSH, T3, T4 and Tg levels, and follow-up intervals.

We assessed the quality of the included studies in terms of risk of bias using the Cochrane Collaboration’s tool [19].

### Data analysis

RevManManger 5.3 was used to analyze the data. We compared observed indices including nodule volume, TSH, T3, T4 and Tg levels both before and after PLA. For all analyses, *P <* 0.05 was considered statistically significant and indicates that the use of PLA in treating benign TNs was effective.

Heterogeneity affects the accuracy of estimation and was assessed by using chi-square testing and I^2^ statistics [20, 21]. When the *P* value of the chi-square test is greater than 0.1, it indicates that no heterogeneity exists in the included studies. When the *P* value of the chi-square test is less than or equal to 0.1, the I^2^ statistic is applied to further assess heterogeneity. When I^2^ ≥ 25%, it indicates low heterogeneity. When I^2^ ≥ 50%, it indicates moderate heterogeneity. When I^2^ ≥ 75%, it indicates significant heterogeneity.

We summarized the data using a fixed-effect model in the case of no or non-significant heterogeneity, otherwise, in the case of significant heterogeneity, a random-effects model was used [22, 23]. Subgroup analysis or meta-regression were then performed to explore the source of heterogeneity according to the basic characteristics of the included studies including the continent (Asia/Africa or Europe), study design (prospective or retrospective), type of TN (functional or non-functional) and energy used (less than 1000J, between 1000J-2000J, between 2000J-3000J, or more than 3000J). In addition, a sensitivity analysis was used to estimate heterogeneity by removing the effect of the larger studies and reevaluating the heterogeneity of the included studies. Finally, publication bias was evaluated using funnel plots and Egger’s linear regression [24]. When a funnel plot is asymmetrical, interpretation of the results should be assessed critically. Otherwise, no publication bias exists.

## SUPPLEMENTARY MATERIALS TABLE





## References

[1] Gharib H, Papini E (2007). Thyroid nodules: clinical importance, assessment, and treatment. Endocrinol Metab Clin North Am.

[2] Gharib H, Papini E, Paschke R, Duick DS, Valcavi R, Hegedüs L, Vitti P, AACE/AME/ETA Task Force on Thyroid Nodules (2010). American Association of Clinical Endocrinologists, Associazione Medici Endocrinologi, and European Thyroid Association Medical guidelines for clinical practice for the diagnosis and management of thyroid nodules: executive summary of recommendations. Endocr Pract.

[3] Arora N, Scognamiglio T, Zhu B, Fahey TJ (2008). Do benign thyroid nodules have malignant potential? An evidence-based review. World J Surg.

[4] Miccoli P, Minuto MN, Ugolini C, Pisano R, Fosso A, Berti P (2008). Minimally invasive video-assisted thyroidectomy for benign thyroid disease: an evidence-based review. World J Surg.

[5] Hegedüs L (2004). Clinical practice. The thyroid nodule. N Engl J Med.

[6] Jeong WK, Baek JH, Rhim H, Kim YS, Kwak MS, Jeong HJ, Lee D (2008). Radiofrequency ablation of benign thyroid nodules: safety and imaging follow-up in 236 patients. Eur Radiol.

[7] Bandeira-Echtler E, Bergerhoff K, Richter B (2014). Levothyroxine or minimally invasive therapies for benign thyroid nodules. Cochrane Database Syst Rev.

[8] Kim SM, Baek JH, Kim YS, Sung JY, Lim HK, Choi H, Lee JH (2011). Efficacy and safety of ethanol ablation for thyroglossal duct cysts. AJNR Am J Neuroradiol.

[9] Sung JY, Kim YS, Choi H, Lee JH, Baek JH (2011). Optimum first-line treatment technique for benign cystic thyroid nodules: ethanol ablation or radiofrequency ablation?. AJR Am J Roentgenol.

[10] Rojeski MT, Gharib H (1985). Nodular thyroid disease. Evaluation and management. N Engl J Med.

[11] Kim YJ, Baek JH, Ha EJ, Lim HK, Lee JH, Sung JY, Kim JK, Kim TY, Kim WB, Shong YK (2012). Cystic versus predominantly cystic thyroid nodules: efficacy of ethanol ablation and analysis of related factors. Eur Radiol.

[12] Baek JH, Ha EJ, Choi YJ, Sung JY, Kim JK, Shong YK (2015). Radiofrequency versus ethanol ablation for treating predominantly cystic thyroid nodules: a randomized clinical trial. Korean J Radiol.

[13] Yoon HM, Baek JH, Lee JH, Ha EJ, Kim JK, Yoon JH, Kim WB (2014). Combination therapy consisting of ethanol and radiofrequency ablation for predominantly cystic thyroid nodules. AJNR Am J Neuroradiol.

[14] Chen F, Tian G, Kong D, Zhong L, Jiang T (2016). Radiofrequency ablation for treatment of benign thyroid nodules: A PRISMA-compliant systematic review and meta-analysis of outcomes. Medicine (Baltimore).

[15] Ha EJ, Baek JH, Kim KW, Pyo J, Lee JH, Baek SH, Døssing H, Hegedüs L (2015). Comparative efficacy of radiofrequency and laser ablation for the treatment of benign thyroid nodules: systematic review including traditional pooling and bayesian network meta-analysis. J Clin Endocrinol Metab.

[16] Papini E, Rago T, Gambelunghe G, Valcavi R, Bizzarri G, Vitti P, De Feo P, Riganti F, Misischi I, Di Stasio E, Pacella CM (2014). Long-term efficacy of ultrasound-guided laser ablation for benign solid thyroid nodules. Results of a three-year multicenter prospective randomized trial. J Clin Endocrinol Metab.

[17] Filetti S, Durante C, Torlontano M (2006). Nonsurgical approaches to the management of thyroid nodules. Nat Clin Pract Endocrinol Metab.

[18] Liberati A, Altman DG, Tetzlaff J, Mulrow C, Gøtzsche PC, Ioannidis JP, Clarke M, Devereaux PJ, Kleijnen J, Moher D (2009). The PRISMA statement for reporting systematic reviews and meta-analyses of studies that evaluate health care interventions: explanation and elaboration. Ann Intern Med.

[19] Higgins JP, Altman DG, Gøtzsche PC, Jüni P, Moher D, Oxman AD, Savovic J, Schulz KF, Weeks L, Sterne JA, Cochrane Bias Methods Group, Cochrane Statistical Methods Group (2011). The Cochrane Collaboration’s tool for assessing risk of bias in randomised trials. BMJ.

[20] Higgins JP, Thompson SG (2002). Quantifying heterogeneity in a meta-analysis. Stat Med.

[21] Higgins JP, Thompson SG, Deeks JJ, Altman DG (2003). Measuring inconsistency in meta-analyses. BMJ.

[22] Wood L, Egger M, Gluud LL, Schulz KF, Jüni P, Altman DG, Gluud C, Martin RM, Wood AJ, Sterne JA (2008). Empirical evidence of bias in treatment effect estimates in controlled trials with different interventions and outcomes: meta-epidemiological study. BMJ.

[23] Higgins JP, Thompson SG, Spiegelhalter DJ (2009). A re-evaluation of random-effects meta-analysis. J R Stat Soc Ser A Stat Soc.

[24] Egger M, Davey Smith G, Schneider M, Minder C (1997). Bias in meta-analysis detected by a simple, graphical test. BMJ.

[25] Døssing H, Bennedbaek FN, Karstrup S, Hegedüs L (2002). Benign solitary solid cold thyroid nodules: US-guided interstitial laser photocoagulation—initial experience. Radiology.

[26] Spiezia S, Vitale G, Di Somma C, Pio Assanti A, Ciccarelli A, Lombardi G, Colao A (2003). Ultrasound-guided laser thermal ablation in the treatment of autonomous hyperfunctioning thyroid nodules and compressive nontoxic nodular goiter. Thyroid.

[27] Pacella CM, Bizzarri G, Spiezia S, Bianchini A, Guglielmi R, Crescenzi A, Pacella S, Toscano V, Papini E (2004). Thyroid tissue: US-guided percutaneous laser thermal ablation. Radiology.

[28] Papini E, Guglielmi R, Bizzarri G, Pacella CM (2004). Ultrasound-guided laser thermal ablation for treatment of benign thyroid nodules. Endocr Pract.

[29] Døssing H, Bennedbaek FN, Hegedüs L (2005). Effect of ultrasound-guided interstitial laser photocoagulation on benign solitary solid cold thyroid nodules - a randomised study. Eur J Endocrinol.

[30] Cakir B, Topaloglu O, Gul K, Agac T, Aydin C, Dirikoc A, Gumus M, Yazicioglu K, Ersoy RU, Ugras S (2006). Effects of percutaneous laser ablation treatment in benign solitary thyroid nodules on nodule volume, thyroglobulin and anti-thyroglobulin levels, and cytopathology of nodule in 1 yr follow-up. J Endocrinol Invest.

[31] Døssing H, Bennedbaek FN, Hegedüs L (2006). Effect of ultrasound-guided interstitial laser photocoagulation on benign solitary solid cold thyroid nodules: one versus three treatments. Thyroid.

[32] Barbaro D, Orsini P, Lapi P, Pasquini C, Tuco A, Righini A, Lemmi P (2007). Percutaneous laser ablation in the treatment of toxic and pretoxic nodular goiter. Endocr Pract.

[33] Døssing H, Bennedbaek FN, Bonnema SJ, Grupe P, Hegedüs L (2007). Randomized prospective study comparing a single radioiodine dose and a single laser therapy session in autonomously functioning thyroid nodules. Eur J Endocrinol.

[34] Papini E, Guglielmi R, Bizzarri G, Graziano F, Bianchini A, Brufani C, Pacella S, Valle D, Pacella CM (2007). Treatment of benign cold thyroid nodules: a randomized clinical trial of percutaneous laser ablation versus levothyroxine therapy or follow-up. Thyroid.

[35] Cakir B, Ugras NS, Gul K, Ersoy R, Korukluoglu B (2009). Initial report of the results of percutaneous laser ablation of benign cold thyroid nodules: evaluation of histopathological changes after 2 years. Endocr Pathol.

[36] Valcavi R, Riganti F, Bertani A, Formisano D, Pacella CM (2010). Percutaneous laser ablation of cold benign thyroid nodules: a 3-year follow-up study in 122 patients. Thyroid.

[37] Døssing H, Bennedbæk FN, Hegedüs L (2011). Long-term outcome following interstitial laser photocoagulation of benign cold thyroid nodules. Eur J Endocrinol.

[38] Gambelunghe G, Fede R, Bini V, Monacelli M, Avenia N, D’Ajello M, Colella R, Nasini G, De Feo P (2013). Ultrasound-guided interstitial laser ablation for thyroid nodules is effective only at high total amounts of energy: results from a three-year pilot study. Surg Innov.

[39] Gambelunghe G, Bini V, Stefanetti E, Colella R, Monacelli M, Avenia N, De Feo P (2014). Thyroid nodule morphology affects the efficacy of ultrasound-guided interstitial laser ablation: a nested case-control study. Int J Hyperthermia.

[40] Pacella CM, Mauri G, Achille G, Barbaro D, Bizzarri G, De Feo P, Di Stasio E, Esposito R, Gambelunghe G, Misischi I, Raggiunti B, Rago T, Patelli GL (2015). Outcomes and risk factors for complications of laser ablation for thyroid nodules: a multicenter study on 1531 patients. J Clin Endocrinol Metab.

[41] Achille G, Zizzi S, Di Stasio E, Grammatica A, Grammatica L (2016). Ultrasound-guided percutaneous laser ablation in treating symptomatic solid benign thyroid nodules: our experience in 45 patients. Head Neck.

[42] Mauri G, Cova L, Monaco CG, Sconfienza LM, Corbetta S, Benedini S, Ambrogi F, Milani V, Baroli A, Ierace T, Solbiati L (2016). Benign thyroid nodules treatment using percutaneous laser ablation (PLA) and radiofrequency ablation (RFA). Int J Hyperthermia.

[43] Negro R, Salem TM, Greco G (2016). Laser ablation is more effective for spongiform than solid thyroid nodules. A 4-year retrospective follow-up study. Int J Hyperthermia.

[44] Ritz JP, Isbert C, Roggan A, Germer CT, Albrecht D, Buhr HJ (1998). Correlation of intrahepatic light and temperature distribution in laser-induced thermotherapy of liver tumors and liver tissue. Gastroenterology.

[45] Albrecht D, Germer CT, Roggan A, Isbert C, Ritz JP, Buhr HJ (1998). [Laser-induced thermotherapy. Technical prerequisites for treatment of malignant liver tumors]. [Article in German]. Chirurg.

[46] Ritz JP, Lehmann KS, Zurbuchen U, Knappe V, Schumann T, Buhr HJ, Holmer C (2009). *Ex vivo* and *in vivo* evaluation of laser-induced thermotherapy for nodular thyroid disease. Lasers Surg Med.

[47] Ritz JP, Lehmann KS, Schumann T, Knappe V, Zurbuchen U, Buhr HJ, Holmer C (2011). Effectiveness of various thermal ablation techniques for the treatment of nodular thyroid disease—comparison of laser-induced thermotherapy and bipolar radiofrequency ablation. Lasers Med Sci.

[48] Spencer CA, Wang CC (1995). Thyroglobulin measurement. Techniques, clinical benefits, and pitfalls. Endocrinol Metab Clin North Am.

[49] Spiezia S, Garberoglio R, Milone F, Ramundo V, Caiazzo C, Assanti AP, Deandrea M, Limone PP, Macchia PE, Lombardi G, Colao A, Faggiano A (2009). Thyroid nodules and related symptoms are stably controlled two years after radiofrequency thermal ablation. Thyroid.

[50] Mauri G, Cova L, Ierace T, Baroli A, Di Mauro E, Pacella CM, Goldberg SN, Solbiati L (2016). Treatment of metastatic lymph nodes in the neck from papillary thyroid carcinoma with percutaneous laser ablation. Cardiovasc Intervent Radiol.

[51] Burke DR, Lewis CA, Cardella JF, Citron SJ, Drooz AT, Haskal ZJ, Husted JW, McCowan TC, Van Moore A, Oglevie SB, Sacks D, Spies JB, Towbin RB, Bakal CW, Society of Interventional Radiology Standards of Practice Committee (2003). Quality improvement guidelines for percutaneous transhepatic cholangiography and biliary drainage. J Vasc Interv Radiol.

[52] Lewis CA, Allen TE, Burke DR, Cardella JF, Citron SJ, Cole PE, Drooz AT, Drucker EA, Haskal ZJ, Martin LG, Van Moore A, Neithamer CD, Oglevie SB, Society of Interventional Radiology Standards of Practice Committee (2003). Quality improvement guidelines for central venous access. J Vasc Interv Radiol.

[53] Rozenblum N, Zeira E, Scaiewicz V, Bulvik B, Gourevitch S, Yotvat H, Galun E, Goldberg SN (2015). Oncogenesis: an “off-target” effect of radiofrequency ablation. Radiology.

[54] Velez E, Goldberg SN, Kumar G, Wang Y, Gourevitch S, Sosna J, Moon T, Brace CL, Ahmed M (2016). Hepatic thermal ablation: effect of device and heating parameters on local tissue reactions and distant tumor growth. Radiology.

