# Supplementary material for: Percutaneous laser ablation for benign thyroid nodules: a meta-analysis
Source: Oncotarget. 2017 May 17;8(47):83225–36. doi: 10.18632/oncotarget.17928 (PMC5669962; doi:10.18632/oncotarget.17928)
Supplement: Supplementary file 1 [file oncotarget-08-83225-s001.pdf]

Percutaneous laser ablation for benign thyroid nodules: a meta-analysis

Supplementary Materials

Supplementary Table 1: The characteristic of included studies. See Supplementary_Table_1
