# Supplementary material for: Percutaneous laser ablation for benign thyroid nodules: a meta-analysis
Source: Oncotarget. 2017 May 17;8(47):83225–36. doi: 10.18632/oncotarget.17928 (PMC5669962; doi:10.18632/oncotarget.17928)
Supplement: Supplementary file 2 [file oncotarget-08-83225-s002.docx]

| Author | Year | Nation | Design | Patients  (male/female) | Age, y | Nodules  characteristics | Laser energy /times | Observed Index | Follow-up Interval(month) |
| --- | --- | --- | --- | --- | --- | --- | --- | --- | --- |
| Helle Døssing | 2002 | Denmark | prospective | 16(3/13) | 47 | solitary solid benign TNs | 761J/1 | Nodule volume | 6 |
| Stefano Spiezia | 2003 | Italy | NG | 12(1/11) | 53.8 | autonomously hyperfunctioning TNs nodular goiter | 1800 J/1 | Nodule volume | 3,12 |
| C. M. Pacella | 2004 | Italy | NG | 25(3/22) | 54.2 | cold benign and autonomously hyperfunctioning TNs | 10150J/1  4200J/11 | Nodule volume | 6 |
| Enrico Papini | 2004 | Italy | NG | 20(5/15) | 63.3 | hypofunctioning benign TNs | 1800 J/1 | Nodule volume,T3,T4,TSH, TPOAb | 6 |
| Helle Døssing | 2005 | Denmark | prospective | 15(0/15) | 47 | solitary solid and cold benign TNs | 2007J/1 | Nodule volume,T3,T4,TSH, Anti-TPOAb | 1,2,6 |
| Bekir Cakir | 2006 | Turkey | NG | 12(4/8) | 47.2 | solitary solid benign TNs | 2725J/1,4,5 | Nodule volume,T3,T4,TSH, Anti-TPOAb, Anti-TPO | 6,9,12 |
| Helle Døssing | 2006 | Denmark | prospective | 15(1/14)  15(0/15) | 46  45 | solitary solid and cold benign TNs | 2007J /1,3 | Nodule volume,T3,T4,TSH, Anti-TPOAb | 1,2,6 |
| Daniele Barbaro | 2007 | Italy | NG | 18(8/10) | 31-80 | Toxic and pre-toxic nodular goiter | NG | T3,T4,TSH, | 1,2,3,12 |
| Enrico Papini | 2007 | Italy | NG | 21(3/18) | 44.9 | single or dominant TNs | 1504J/1 | Nodule volume,T3,T4,TSH, Tg, TPOAb, TgAb | 12 |
| Helle Døssing | 2007 | Denmark | prospective | 14(3/11) | 58 | solitary hot TNs | NG | Nodule volume,T3,T4,TSH, Anti-TPOAb | 1,3,6 |
| Bekir Cakir | 2009 | Turkey | NG | 3(1/2) | 51 | cold benign TNs | 2740J/1 | Nodule volume,T3,T4,TSH, Anti-TPOAb, Anti-TPO,Tg, Anti-Tg | 1,3,6,12,24 |
| Roberto Valcavi | 2010 | Italy | retrospective | 122(27/95) | 52.2 | solitary cold benign TNs | 8522J/1 | Nodule volume,T3,T4,TSH, Tg | 1,6,12,24,36 |
| Helle Døssing | 2011 | Denmark | NG | 78(4/74) | 46 | solitary solid and cold benign TNs | 2100J/1 | Nodule volume | 12 |
| Giovanni Gambelunghe | 2012 | Italy | retrospective | 40(NG) | 62/64 | solitary solid and cold benign TNs | 71.5 J/1  578.5 J/1 | Nodule volume, T4,TSH, Tg | 3,6,12,24,36 |
| Giovanni Gambelunghe | 2014 | Italy | NG | 20(7/13) | 56 | Single and fusion multiple benign TNs, | 378J/1 | Nodule volume | 6 |
| C. M. Pacella | 2015 | Italy | retrospective | 1531(444/1087) | 54.1 | solitary solid benign TNs | 1200-1800/1 | Nodule volume | 12 |
| Gaetano Achille | 2016 | Italy | retrospective | 45(10/35) | 52 | solid benign TNs | 1304 J/1 | Nodule volume | 6,12 |
| Giovanni Mauri | 2016 | Italy | retrospective | 59(11/48) | 55.2 | Benign TNs | 5422.2J/1 | Nodule volume | 1,6,12 |
| Roberto Negro | 2016 | Egypt | retrospective | 56(14/42) | 54.7 | solitary solid benign TNs | 6168J/1 | Nodule volume,T3,T4,TSH, Tg | 3,6,12,24,36,48 |

Supplementary Table.1 The characteristic of included studies
